# Correction: Innovative wound management: creating dynamic Alg-Mg/SF hydrogels for controlled Mg^2+^ release in wound healing

**DOI:** 10.1039/d4ra90057j

**Published:** 2024-05-21

**Authors:** Chaolun Dai, Bingxin Wu, Min Chen, Yisheng Gao, Miao Zhang, Wanhua Li, Guicai Li, Qinzhi Xiao, Yahong Zhao, Yumin Yang

**Affiliations:** a Key Laboratory of Neuroregeneration of Jiangsu and Ministry of Education, Co-innovation Center of Neuroregeneration, NMPA Key Laboratory for Research and Evaluation of Tissue Engineering Technology Products, Nantong University Nantong 226001 P. R. China; b Medical School, Nantong University Nantong 226001 P. R. China; c Department of Echocardiography Centre, Affiliated Hospital of Nantong University 226001 Nantong P. R. China; d Department of Pediatrics, Affiliated Hospital of Nantong University 226001 Nantong P. R. China

## Abstract

Correction for ‘Innovative wound management: creating dynamic Alg-Mg/SF hydrogels for controlled Mg^2+^ release in wound healing’ by Chaolun Dai *et al.*, *RSC Adv.*, 2024, **14**, 10874–10883, https://doi.org/10.1039/D4RA00793J.

The authors regret that the name of one of the authors (Bingxin Wu) was shown incorrectly in the original article. The corrected author list is as shown here.

The Royal Society of Chemistry apologises for these errors and any consequent inconvenience to authors and readers.

## Supplementary Material

